# Ruling Out Brain CT Contraindications prior to Intravenous Thrombolysis: Diagnostic Equivalence between a Primary Interpretation Workstation and a Mobile Tablet Computer

**DOI:** 10.1155/2017/6869145

**Published:** 2017-11-09

**Authors:** Antonio J. Salazar, Nicolás Useche, Manuel Granja, Aníbal J. Morillo, Sonia Bermúdez

**Affiliations:** ^1^Electrophysiology and Telemedicine Laboratory, University of Los Andes, Carrera 1 Este No. 19A-40, Bogotá, Colombia; ^2^Primary Stroke Center, University Hospital of Fundación Santa Fe de Bogotá, Calle 119 No. 7-75, Bogotá, Colombia; ^3^School of Medicine, Universidad El Bosque, Av. Cra 9 No. 131A-02, Bogotá, Colombia; ^4^Baptist Health, Lyerly Neurosurgery, 800 Prudential Drive, Jacksonville, FL 32207, USA

## Abstract

**Objective:**

The aim of this study was to evaluate the equivalence of brain CT interpretations performed using a diagnostic workstation and a mobile tablet computer, in a telestroke service.

**Materials and Methods:**

The ethics committee of our institution approved this retrospective study. A factorial design with 1452 interpretations was used. The assessed variables were the type of stroke classification, the presence of contraindications to the tPA administration, the presence of a hyperdense intracranial artery sign (HMCA), and the Alberta Stroke Program Early CT Score (ASPECTS) score. These variables were evaluated to determine the effect that the reading system had on their magnitudes.

**Results:**

The achieved distribution of observed lesions using both the reading systems was not statistically different. The differences between the two reading systems to claim equivalence were 1.6% for hemorrhagic lesions, 4.5% for cases without lesion, and 5.2 for overall ischemic lesion. Equivalence was achieved at 2.1% for ASPECTS ≤ 6, 6.5% for the presence of imaging contraindication to the tPA administration, and 7.2% for the presence of HMCA.

**Conclusion:**

The diagnostic performance for detecting acute stroke is likely equivalent whether a tablet computer or a diagnostic workstation is used or not.

## 1. Introduction

A stroke is an acute neurologic dysfunction of vascular origin that involves focal areas in the brain and is a major cause of death and disability in developed [1–3] and developing countries [4] such as ours (Colombia) [5–7]. A stroke can be ischemic (near 87% of cases) or hemorrhagic, and both require different treatments and neurological and radiological expertise for diagnosis. For ischemic stroke, the early administration of tissue plasminogen activator (tPA) reduces disability, as measured by the modified Rankin scale (mRs) at 90 days, but only if it is administered before 4.5 hours [8]. However, even in countries such as the US, only 5% of eligible patients receive this treatment [8] due to geographic distances to primary stroke centers and the limited availability of vascular neurologists and neuroradiologist that after a complete neurological and radiological assessment define if a patient is eligible to receive this treatment. Noncontrast brain computed tomography (CT) is the most widely used method for the first imaging examination in patients with acute stroke symptoms [9].

The aim of this study was to evaluate the diagnostic equivalence of brain CT interpretations when using a primary diagnostic interpretation workstation and a mobile tablet computer in an emergency telestroke setting after providing radiologists with real clinical scenarios. The assessed variables were the distribution of the type of stroke classification, the detection of the presence of any imaging contraindication to the tPA administration for acute ischemic lesions, confidence in the presence of a hyperdense intracranial artery sign (HMCA), the Alberta Stroke Program Early CT Score (ASPECTS) [10], and the reading time. These variables were evaluated to determine the effect that the reading system had on their magnitudes as well as the variables' equivalence between the reading systems.

We were interested in evaluating mobile solutions to be used for telestroke at our hospital. By means of real clinical scenarios that included a complete clinical background, specific neurological symptoms, and the time of symptoms onset, we designed this study to perform image interpretations as closely and realistically as in routine clinical practice, using, first, emergency brain CT images instead of computer tomography angiography (CTA). We used the same radiologists who routinely read brain CT images in our hospital (i.e., neuroradiologists and neuroradiology fellows), and the same clinical information provided to radiologists in clinical practice (e.g., admission diagnosis, neurological symptoms, age, and sex). In addition, the same interpretation process was conducted, in which the type of stroke (i.e., hemorrhagic lesion, acute ischemic lesion, chronic ischemic lesion, or without lesion) was classified. Furthermore, according to the selected lesion type, the radiologist classified other variables: the presence of any imaging contraindication to the tPA administration; confidence in the presence of the hyperdense middle cerebral artery sign (HMCA); and the ASPECTS score (ranging from 1–10), which is a method for assessing the size of an infarct on CT scans in patients with acute stroke to determine when to administer the tPA treatment. Patients with ASPECTS scores less than or equal to six or with the presence of any other imaging contraindication for the tPA administration are not eligible to receive intravenous tPA; therefore, it is critical to detect these group of patients by means of brain CT scans. Although previous studies [9, 11–15] have evaluated the potential equivalence between diagnostic workstations and mobile tablet computers for the interpretation of stroke brain CT scans they lack the specific statistical methods used here along with the selected variables considered in our paper. Scoring variables like the ASPECTS were obtained after providing radiologists with a complete clinical background of anonymous patients and also considering common imaging CT contraindications for tPA administration. This whole approach resembles a real-life scenario just like the diagnostic process of any acute stroke patient.

No significant differences have been reported to support the null hypothesis that the performances of different reading methods are equal and because the power test was not reported, it is not clear if these studies failed to find significant differences given that failure to reject the null hypothesis does not mean this hypothesis is necessary true. In contrast, the present study was set to evaluate equivalence or noninferiority based on significant results. To establish the fact that the performances are equal or that one modality is noninferior to the other, the null hypothesis has to be that their performances are not equal or that one is inferior to the other. Only by rejecting such a hypothesis can it be concluded that the modalities under comparison are equivalent [16–19].

## 2. Materials and Methods

This retrospective study was approved by the ethics committee of our institution, and informed consent was not required. We employed a factorial design with repeated measures.

### 2.1. Sample

Patients with symptoms of acute stroke, who presented to the emergency room for acute stroke evaluation between 2013 and 2016 at the Blinded University Hospital (Blinded), were included in the study. Our hospital is a primary Joint Commission International- (JCI-) certified stroke center with endovascular treatment capabilities. The patients were randomly selected without repetition. Cases with image artifacts were excluded. The cases consisted of brain CT examinations stored in our hospital PACS, which were acquired using a General Electric LightSpeed 64 slice CT scanner (General Electric Healthcare, GE Medical Systems, Milwaukee, WI, USA), with 100 kV, 10 mAs, axial: 5 mm, sagittal: 3 mm, FOV: 26 cm, pixel spacing: 0.469, matrix: 512 × 512, and pitch: 0.984:1.

Based on a neuroradiologist routine primary diagnostic interpretation, the sample included patients without lesions (7) and those with hemorrhagic lesions (11), acute ischemic lesions (67), and chronic ischemic lesions (36). The ages ranged from 30 to 97 years, with a mean age of 70.8 years (standard deviation of 15.2). There were 59 males and 62 females.

### 2.2. Observers and Reading Observed Variables

Five neuroradiologists (three with more than ten years of experience) and one neuroradiology fellow were selected as observers. They were asked to classify the type of stroke (i.e., hemorrhagic lesion, acute ischemic lesion, chronic ischemic lesion, or without lesion). For acute ischemic lesions, the radiologists classified variables that included contraindications to the tPA administration for acute ischemic lesions (e.g., subacute ischemic stroke, intra-axial neoplasm, arteriovenous malformation, aneurysm, hemorrhagic transformation of an ischemic infarct, and hypodensity > 1/3 of the middle cerebral artery vascular territory) and the confidence in the presence of a hyperdense middle cerebral artery (HMCA) from the following scores: 0: definitely absent; 1: most likely absent; 2: cannot decide; 3: most likely present; and 4: definitely present. Finally, the ASPECTS score (ranged from 1 to 10) was recorded.

### 2.3. Display Monitors and Viewer Software

According to the American College of Radiology (ACR) guidelines for teleradiology [20], small matrix size images, such as CT, must be visualized on a monitor with a minimum of 512 × 512 matrix size at a minimum 8-bit pixel depth, for processing or manipulation with no loss of matrix size or bit depth at display. In addition, the Digital Imaging and Communications in Medicine (DICOM) standard recommends the use of monitors calibrated to a maximum luminance of 400–500 cd/m^2^ and calibrated according to the DICOM grayscale standard display function (GSDF) [21]. Therefore, we used two displays with these specifications.

The routine system for CT readings in our hospital is a PACS workstation with a DICOM-compliant viewer software Agfa IMPAX 6.5 (AGFA HealthCare, Mortsel, Belgium). Images were displayed using an E-2620 BARCO monitor (BARCO N.V, Kortrijk, Belgium), which is a 2-megapixel (MPx) LCD medical grayscale display, DICOM-compliant, dot pitch of 0.249 mm, with a spatial resolution of 1600 × 1200 pixels, a maximum luminance of 700 cd/m^2^, and an 8-bit grayscale. This system, hereafter referred to as MEDICAL-IMPAX, was used as the reference reading system in this study.

For the mobile option, an Apple iPad Pro 9.7 MLMN2CL/A (Apple Inc., Cupertino, CA, USA), with a “retina” display of 9.7 inches, dot pitch of 0.096 mm (264 dpi), with a spatial resolution of 1536 × 2048 pixels, and a maximum luminance of 500 cd/m^2^ was selected. The viewer used on this tablet was the Agfa XERO Viewer 3.0 (Agfa HealthCare, Mortsel, Belgium) software, hereafter referred to as TABLET-XERO.

### 2.4. Procedure

Each radiologist read all cases using both the Medical-IMPAX and the Tablet-XERO systems. At each reading, the radiologist determined the variables mentioned in the section “observers and reading observed variables.” The two reading software packages provided image manipulation tools to adjust window/level, zoom, and multiplanar reformation presentation. These tools were available for all images and could be used at the observer's discretion to improve the image interpretations. For each reading session, the radiologist verified the settings of the contrast and luminance of the display with the RP-133 pattern in a controlled illumination (ambient light of approximately 20 lux according to the American Association of Physicists in Medicine [AAPM] TG18 recommendations) [22].

The radiologists were blinded to the patient and examination identification, to the original interpretation, and to the type of lesion. To ensure that the radiologists were blinded, a junior radiology resident was in charge of uploading the images into the PACS workstation or tablet using the IMPAX or XERO viewer software. Data collection was performed using a web-based form, and the interpretations were stored in a database. This software presents the patients to be interpreted at random and guides the radiologist to complete the report, thus ensuring the integrity and completeness of data.

According to Mullins et al., the availability of a clinical history consistent with an early stroke significantly improves the sensitivity for detecting strokes on unenhanced brain CT. Thus, whenever possible, relevant clinical history should be made available to the physicians interpreting emergency CT scans of the head [23]. Therefore, the clinical history that is used in the standard protocol to interpret brain CT with suspicion of acute stroke was available to the radiologists, including the sex, age, main neurological symptoms, and relevant past medical history (e.g., diabetes, hypertension, headache, Parkinson, Alzheimer, sleep apnea/hypopnea syndrome, or cardiac arrhythmia). This was done to achieve a more realistic interpretation scenario. Hence, the only difference from the real practice was the reading system. All information was presented on a web-based collection form before beginning each image interpretation.

There was at least a five-month interval between the readings from the same patient by the same radiologist using the compared systems. The readings were performed over the course of ten months in two- or four-hour sessions by each radiologist, with no time limitations for each reading. The initial display used the default image window setting (WW = 174 and WL = 55), but the radiologists were free to select another window, such as a cerebral or a stroke window (WW = 80 and WL = 40, WW = 40 and WL = 40, resp.).

### 2.5. Data Analysis

Patients with an ASPECTS ≤ 6 were not eligible to receive the tPA treatment. Therefore, we dichotomized the ASPECTS score into two categories: 1 if the score ranged from 1 to 6 (a contraindication for tPA administration from the imaging point of view) and 0 if the score ranged from 7 to 10 (indicating eligibility for the administration of the tPA treatment from the imaging point of view). This variable was named “ASPECTS ≤ 6.”

The variable confidence in the presence of a hyperdense middle cerebral artery (HMCA) was also dichotomized as follows: 0 if the score ranged 0–1 (HMCA not detected) and 1 if the score ranged from 2 to 4 (HMCA detected). This variable was named “presence of HMCA.”

We evaluated the diagnostic equivalence between the reading systems based on (1) the distribution of type of stroke classification, (2) the “ASPECTS ≤ 6,” (3) the detection of the presence of any imaging contraindication to the tPA administration for acute ischemic lesions, (4) the “presence of HMCA,” and (5) the reading time.

These variables and the difference between the systems were evaluated using generalized estimating equations (GEE) with the IBM SPSS Statistics 19 software (IBM Corp., Armonk, NY, USA). To evaluate noninferiority and equivalence, the mean differences and their standard errors were obtained from the GEE analysis.

The hypothesis test for equivalence was as follows: the null hypothesis Ho was |Mean  Difference  (*I* − *J*)| − *δ* = 0, and the alternative hypothesis Ha was |Mean  Difference  (*I* − *J*)| − *δ* < 0, where *I* and *J* are the two reading systems compared and *δ* (delta) is the maximum allowable difference permitted to conclude equivalence or noninferiority, as suggested by several authors in recent years [16–19]. We calculated a (1 − 2*α*)% confidence interval for all comparisons, which is a method to evaluate equivalence [18, 19]. The significance level was set to 5% (i.e., *α* = 0.05), and *δ* was set to 0.1 (10%) for proportional variables and 15 seconds for reading time. Finally, we calculated the required value of *δ* to claim equivalence for each variable (named *δ*_eq_ in our result in Tables 2 and 4).

## 3. Results

### 3.1. Distribution of Type of Stroke Classification

The distribution of the type of stroke classification for each reading system and their comparisons are shown in Table 1. The observed category distribution of cases (i.e., without lesion, hemorrhagic lesion, acute ischemic lesion, and chronic ischemic lesion), using both Medical-IMPAX and Tablet-XERO, was not significantly different (*P* = 0.332). To compare the number of cases detected by a reading system in each lesion category, nonsignificant differences were observed for hemorrhagic lesion (*P* = 0.155) and without lesions (*P* = 0.194). Although significant differences were observed for acute and chronic ischemic lesions, nonsignificant differences were noted for the overall ischemic lesions (combining acute and chronic ischemic lesions).

### 3.2. Equivalence Tests for the Distribution on the Type of Stroke Classification

Equivalence tests for the distribution on the type of stroke classification between the two reading systems are shown in Table 2. For *δ* (delta) values of equivalence of 0.1 (i.e., 10%) to claim equivalence, both reading systems were equivalent (all *P* < 0.05) for all individual type of stroke classification categories, even for chronic ischemic lesions.

In addition, we performed a sensitivity analysis for the equivalence *δ* value to determine the *δ*_eq_ value for which equivalence may be claimed with significant tests. In this analysis, Medical-IMPAX and Tablet-XERO achieved equivalence at 4.5% for the category without lesions, 1.6% for hemorrhagic lesion, 8.7% for acute ischemic lesion, and 4.8% for chronic ischemic lesion. In addition, a value of 5.2% for the overall ischemic lesion was observed.

### 3.3. Diagnostic Variables and Reading Time

The mean values and the comparisons for the proportions observed on the “ASPECTS ≤ 6,” the presence of any imaging contraindication to the tPA administration for acute ischemic lesions, presence of HMCA, and reading time are presented in Table 3. The proportion of patients observed with an ASPECTS ≤ 6 was 0.14 for both reading systems, and nonsignificant differences were observed (*P* = 0.855). Similarly, nonsignificant differences were observed in the proportion of the presence of any imaging contraindication to the tPA administration for acute ischemic lesions (*P* = 0.476), with values of 0.32 on Medical-IMPAX and 0.34 on Tablet-XERO. However, the proportion of cases in which the presence of HMCA was detected using Medical-IMPAX and Tablet-XERO was 0.24 and 0.29, respectively, with significant differences observed (*P* = 0.027).

The reading times for the interpretations on Medical-IMPAX and Tablet-XERO were 126 s and 123 s, respectively, which were not significantly different (*P* = 0.566). The difference in the time to claim equivalence was 13.7 s (*P* < 0.05).

### 3.4. Equivalence Tests for Proportions on the Diagnostic Variables and Reading Time

Equivalence tests for the diagnostic variables and reading time are shown in Table 4. The average reading times for the interpretations on the Medical-IMPAX and Tablet-XERO were 126 s and 123 s, respectively, with no significant difference (*P* = 0.566). For *δ* (delta) values of equivalence of 0.1 (i.e., 10%) to claim equivalence on the diagnostic variables and 15 s on the reading time, both reading systems were equivalent (all *P* < 0.05). In addition, we performed a sensitivity analysis of the equivalence *δ* (delta) value to determine the *δ* value for which equivalence may be claimed with significant values (*δ*_eq_). In this analysis, Medical-IMPAX and Tablet-XERO achieved equivalence at 2.1% for “ASPECTS ≤ 6,” 6.5% for acute ischemic lesion without contraindications, 7.2% for “presence of HMCA,” and 13.7 s for the reading time.

## 4. Discussion

The distribution of observed lesions using both the Medical-IMPAX and the Tablet-XERO was not significantly different. For hemorrhagic lesions and the without lesion categories, differences were also not significantly different. Equivalence for the two reading systems can be claimed for differences of 1.6% for the hemorrhagic lesions. These results for hemorrhagic lesions in our study are in line with those from other studies that reported high agreement and accuracy for identifying hemorrhagic lesions [9, 11, 12]. For overall ischemic lesion (combining acute and chronic ischemic lesions), nonsignificant differences were noted between the two reading systems. The equivalence of the two reading systems can be claimed for differences of 5.2%.

Previous studies have reported nonsignificant differences in the detection of overall ischemic lesion and acute hemorrhage between mobile solutions and PACS workstations [9, 12, 14, 15]. Although the variables and evaluation methods used in these studies were different than those used in ours, this study agrees with the previous works in that the reading system does not affect the detection of hemorrhagic lesions or the overall ischemic lesion.

The results of our study indicate that patients with an ASPECTS ≤ 6 were well detected using both reading systems when the interpretations were performed by experienced radiologists and that the equivalence of the two reading systems may be claimed for low differences (2.1%). When detecting the presence of a HMCA, we observed significant differences. Nevertheless, equivalence was confirmed at differences lower than 10%. Besides, the actual difference achieved to claim equivalence was 7.2%. This assessment was designed to evaluate the equivalence of the magnitudes of the related variables, rather than to find differences or to show that they are equal. In addition, this evaluation is not an accuracy assessment (as the results are not compared to the gold standard). Therefore, it is not possible, at present, to declare which of the two magnitudes is the most accurate. Although more cases with HMCA were detected using the tablet, this does not mean that the tablet was better to detect the HMCA. If the difference is due to true positives, this is a rare result as the tablet has the lower contrast. On the other hand, the difference may be due to false positives, but this is not possible to state at present in the absence of the gold standard, which we will set further.

These findings are consistent with those described by McLaughlin et al., who used the same kind of tablet to determine the interpretations of senior radiologists [9]. To our knowledge, however, no study has described the performance of observers when detecting the presence of any imaging contraindication to the tPA administration for acute ischemic lesions that should not receive tPA treatment. In our study, nonsignificant differences were noted, and equivalence was confirmed at 10%. In addition, the equivalence of the two reading systems may be claimed for differences of 6.5%.

The approach used to perform the interpretations, the assessment process, the variables evaluated, especially, for ruling out brain CT contraindications prior to intravenous thrombolysis, and the statistical methods used to analyze them are the principal differences between our study and previous ones [9, 11–15], which focused on evaluating accuracy or reliability. In contrast, our study was set to evaluate the equivalence of the reading systems on the diagnostic variables (i.e., the distribution of type of stroke classification, the ASPECTS score, the detection of acute ischemic lesions without contraindications, and the confidence in the presence of a hyperdense middle cerebral artery) and on the reading time. In this sense, our study agrees with previous studies in terms of which nonsignificant differences were observed between the reading systems, but, from another perspective, our study investigates the effects of reading systems and the equivalence of the variables assessed. Hence, our study is a complement to the previous studies without statistical type II errors.

At present, we are evaluating other mobile alternatives, such as a smartphone with a high-resolution display and a portable computer, for use by the same observers and in the same sample used in this study. Our three neuroradiologists with more than ten years of experience will develop, by consensus, the reference gold standard for our sample. Nevertheless, to avoid bias, these neuroradiologists will complete all readings before establishing the gold standard. For these reasons, this study is not an accuracy evaluation. Further, when the reference standard is available, we will be able to evaluate the accuracy.

One limitation of this study centers on the illumination conditions. Readings using the Medical-IMPAX system were performed in diagnostic reading stations in which the ambient light levels were controlled according to the AAPM TG18 recommendations (i.e., 15–60 lux) [22]. In contrast, readings using the TABLET-XERO system were performed without controlling ambient light levels. Nevertheless, this situation is more realistic for a telestroke system in which a radiologist is asked to read the brain CT as soon as possible wherever he is located and may reproduce a situation in which a Medical-IMPAX system is not available. The reading time difference was not a concern in this study.

The results of this study provide reassurance to radiologists who interpret brain CTs using the TABLET-XERO system that their performance is likely equivalent to their performance when using the Medical-IMPAX system. To the best of our knowledge, this is the first study that uses nonequivalence or noninferiority statistical methods to identify potential diagnostic differences between a primary diagnostic workstation and a mobile tablet computer in the process of ruling out imaging contraindications prior to intravenous thrombolysis.

In conclusion, the results of the present study show that, even after challenging radiologists with real clinical scenarios of acute stroke patients, mobile solutions with high-resolution displays, such as the “retina” display of the iPAD 3, may provide useful telestroke solutions.

## Figures and Tables

**Table 1:** Comparison of the distribution of the type of stroke classification between the reading systems.

Lesion type	Reading system	Detected cases	Proportion (%)^*∗*^	Std. error	95% Wald confidence		Wald chi-square	*P* value
					Lower	Upper		
Without lesion	Medical-IMPAX	214	29.5	0.032	0.23	0.36	2.024	0.155
	Tablet-XERO	229	31.5	0.032	0.25	0.38		
Hemorrhagic lesion	Medical-IMPAX	64	8.8	0.025	0.04	0.14	1.690	0.194
	Tablet-XERO	69	9.5	0.025	0.05	0.14		
Acute ischemic lesion	Medical-IMPAX	346	47.7	0.035	0.41	0.54	11.051	0.001
	Tablet-XERO	306	42.1	0.035	0.35	0.49		
Chronic ischemic lesion	Medical-IMPAX	102	14.0	0.022	0.10	0.18	6.183	0.013
	Tablet-XERO	122	16.8	0.024	0.12	0.22		
Overall ischemic lesion^†^	Medical-IMPAX	448	61.7	0.036	0.55	0.69	3.428	0.064
	Tablet-XERO	428	59.0	0.035	0.52	0.66		

^**∗**^Each proportion was calculated from 726 readings (121 cases by 6 radiologists). Statistics were evaluated using generalized estimating equations (GEE) analysis. The overall Wald chi-square was 0.941 with a significance of 0.332. ^†^Overall ischemic lesion was calculated as the aggregate values of acute and chronic ischemic lesions.

**Table 2:** Equivalence tests for the distribution on the type of stroke classification between reading systems.

Variable	*δ* (%)	Mean difference^*∗*^	SE	(1 − 2*α*)% CI for equivalence testing		*z*	*P* value (*P* < −*z*)	H	*δ*_eq_ (%)
				Lower	Upper				
Without lesion	10	−0.021	0.015	−0.045	0.003	−5.46	<0.001	Ha	4.5
Hemorrhagic lesion	10	−0.007	0.005	−0.016	0.002	−17.58	<0.001	Ha	1.6
Acute ischemic lesion	10	0.060	0.017	0.033	0.087	−2.41	0.0079	Ha	8.7
Chronic ischemic lesion	10	−0.030	0.011	−0.048	−0.012	−6.32	<0.001	Ha	4.8

Overall ischemic lesion	10	0.028	0.015	0.003	0.052	−4.87	0.0000	Ha	5.2

SE: standard error of the mean difference, CI: confidence interval; Ho: null hypothesis, Ha: alternative hypothesis for testing equivalence, *α*: significance of the test (0.05), *δ*: difference of the means allowed to achieve equivalence, *z*: test for difference of compared devices, that is, *z* = (|Mean  Difference| − *δ*)/SE, H: retained hypothesis equivalence at *δ* level, “Ha” indicates equivalence achieved and “–” indicates fail to reject Ho., *δ*_eq_: *δ* required for equivalence (i.e., *P* < *α*). Ho: |Difference  (*I* − *J*)| − *δ* = 0; Ha: |Difference  (*I* − *J*)| − *δ* < 0. ^*∗*^Each comparison was calculated by six radiologists and two devices.

**Table 3:** Comparison of diagnostic variables and reading time between the reading systems.

Reading system	Readings	Mean^*∗*^	Std. error	95% Wald confidence interval		Wald chi-Square^†^	*P* value
				Lower	Upper		
*ASPECTS ≤ 6 *							
Medical-IMPAX	81/586	0.14	0.027	0.09	0.19	0.034	0.855
Tablet-XERO	84/593	0.14	0.026	0.09	0.19		
*Contraindication to the tPA administration for acute ischemic lesions*							
Medical-IMPAX	112/346	0.32	0.038	0.25	0.40	0.508	0.476
Tablet-XERO	105/306	0.34	0.039	0.27	0.42		
*Presence of HMCA*							
Medical-IMPAX	123/505	0.24	0.029	0.19	0.30	4.912	0.027
Tablet-XERO	141/492	0.29	0.034	0.22	0.35		
*Reading time*							
Medical-IMPAX	726	126.0	4.47	117.1	134.9	0.332	0.566
Tablet-XERO	726	122.5	5.28	112.0	132.9		

ASPECTS = Alberta Stroke Early CT Scan score; HMCA = hyperdense middle cerebral artery; ^**∗**^For ASPECTS ≤ 6, presence of imaging contraindications to the tPA administration, and the presence of HMCA, the mean proportion was calculated from the detected cases on each variable over the number of readings. Statistics were obtained using generalized estimating equations (GEE) analysis; ^†^For the reading time the *F* statistic for ANOVA analysis was used.

**Table 4:** Equivalence tests for the diagnostic variables and reading time.

Variable	*δ*	Mean difference^a^	SE	(1 − 2*α*)% CI for equivalence testing		*z*	*P* value (*P* < −*z*)	H	*δ*_eq_
				Lower	Upper				
ASPECTS ≤ 6	10.0%	−0.002	0.011	−0.021	0.017	−8.59	<0.001	Ha	2.1%
Presence of imaging contraindications to the tPA administration	10.0%	0.020	0.028	−0.026	0.065	−2.90	0.0018	Ha	6.5%
Presence of HMCA	10.0%	−0.040	0.019	−0.072	−0.008	−3.09	0.0010	Ha	7.2%
Reading time	15 s	3.6 s	6.2	−6.6	13.7	−1.85	0.0318	Ha	13.7 s

SE: Standard error of the mean difference, CI: confidence Interval; Ho: null hypothesis, Ha: alternative hypothesis for testing equivalence, *α*: significance of the test (0.05), *δ*: difference of the means allowed to achieve equivalence, *z*: test for difference of compared devices, that is, *z* = (|Mean  Difference| − *δ*)/SE, H: retained hypothesis equivalence at *δ* level, “Ha” indicates equivalence achieved and “–” indicates fail to reject Ho., *δ*_eq_ = *δ* required for equivalence (i.e., *P* < *α*); Ho: |Difference  (*I* − *J*)| − *δ* = 0; Ha: |Difference  (*I* − *J*)| − *δ* < 0. ^a^Each comparison was calculated for six radiologists and two devices.

## References

[1] WHO (1989). WHO Task Force on Stroke and other Cerebrovascular Disorders: stroke Recommendations on stroke prevention, diagnosis and therapy. *Stroke*.

[2] Taylor T. N., Davis P. H., Torner J. C., Holmes J., Meyer J. W., Jacobson M. F. (1996). Lifetime cost of stroke in the United States. *Stroke*.

[3] Bonita R. (1992). Epidemiology of stroke. *The Lancet*.

[4] Saposnik G., Del Brutto O. H. (2003). Stroke in South America: a systematic review of incidence, prevalence, and stroke subtypes. *Stroke*.

[5] Silva F. A., Zarruk J. G., Quintero C. (2006). Enfermedad cerebrovascular en Colombia. *Revista Colombiana de Cardiología*.

[6] Díaz Cabezas R., Ruano Restrepo M. I. (2011). Conocimiento de síntomas y factores de riesgo de enfermedad cerebro vascular en una población urbana colombiana. *Acta Neurológica Colombiana*.

[7] Silva-Sieger F., Arenas-Borda W., Zarruk J. G. (2007). Factores asociados al tiempo de consulta en pacientes con enfermedad cerebrovascular isquémica. *Revista De Neurologia*.

[8] Kulcsar M., Gilchrist S., George M. G. (2014). Improving stroke outcomes in rural areas through telestroke programs: an examination of barriers, facilitators, and state policies. *Telemedicine journal and e-health : the official journal of the American Telemedicine Association*.

[9] McLaughlin P. D., Moloney F., O'Neill S. B. (2017). CT of the head for acute stroke: Diagnostic performance of a tablet computer prior to intravenous thrombolysis. *Journal of Medical Imaging and Radiation Oncology*.

[10] Pexman J. H. W., Barber P. A., Hill M. D. (2001). Use of the Alberta Stroke Program Early CT Score (ASPECTS) for assessing CT scans in patients with acute stroke. *American Journal of Neuroradiology*.

[11] Panughpath S., Kumar S., Kalyanpur A. (2013). Utility of mobile devices in the computerized tomography evaluation of intracranial hemorrhage. *Indian Journal of Radiology and Imaging*.

[12] Tewes S., Rodt T., Marquardt S., Evangelidou E., Wacker F. K., Von Falck C. (2013). Evaluation of the use of a tablet computer with a high-resolution display for interpreting emergency CT scans. *RöFo - Fortschritte auf dem Gebiet der Röntgenstrahlen und der bildgebenden Verfahren*.

[13] Park J. B., Choi H. J., Lee J. H., Kang B. S. (2013). An assessment of the iPad 2 as a CT teleradiology tool using brain CT with subtle intracranial hemorrhage under conventional illumination. *Journal of Digital Imaging*.

[14] Yoshimura K., Nihashi T., Ikeda M. (2013). Comparison of liquid crystal display monitors calibrated with gray-scale standard display function and with *γ* 2.2 and ipad: Observer performance in detection of cerebral infarction on brain ct. *American Journal of Roentgenology*.

[15] Bhatia A., Patel S., Pantol G., Wu Y., Plitnikas M., Hancock C. (2013). Intra and Inter-Observer Reliability of Mobile Tablet PACS Viewer System vs. Standard PACS Viewing Station-Diagnosis of Acute Central Nervous System Events. *Open Journal of Radiology*.

[16] Chen W., Petrick N. A., Sahiner B. (2012). Hypothesis testing in noninferiority and equivalence MRMC ROC studies. *Academic Radiology*.

[17] Jin H., Lu Y. (2009). A non-inferiority test of areas under two parametric ROC curves. *Contemporary Clinical Trials*.

[18] Liu J.-P., Ma M.-C., Wu C.-y., Tai J.-Y. (2006). Tests of equivalence and non-inferiority for diagnostic accuracy based on the paired areas under {ROC} curves. *Statistics in Medicine*.

[19] Obuchowski N. A. (1997). Testing for equivalence of diagnostic tests. *American Journal of Roentgenology*.

[20] ACR American College of Radiology Standard for Teleradiology.

[21] NEMA (2001). Digital imaging and communications in medicine. *Part 14: Grayscale Standrad Display Function*.

[22] Samei E., Badano A., Chakraborty D. (2005). Assessment of display performance for medical imaging systems: Executive summary of AAPM TG18 report. *Medical Physics*.

[23] Mullins M. E., Lev M. H., Schellingerhout D., Koroshetz W. J., Gonzalez R. G. (2002). Influence of availability of clinical history on detection of early stroke using unenhanced CT and diffusion-weighted MR imaging. *American Journal of Roentgenology*.

